# Neutralizing Enterovirus D68 Antibodies in Children after 2014 Outbreak, Kansas City, Missouri, USA

**DOI:** 10.3201/eid2803.211467

**Published:** 2022-03

**Authors:** Robyn A. Livingston, Christopher J. Harrison, Rangaraj Selvarangan

**Affiliations:** Children’s Mercy Hospital Kansas City, Kansas City, Missouri, USA; University of Missouri, Kansas City

**Keywords:** enterovirus, EV-D68, neutralizing antibody, children, seroprevalence, asthma, viruses, B1 clade, B2 clade, D clade, Kansas City, Missouri, USA

## Abstract

Enterovirus D68 (EV-D68) causes severe respiratory illness outbreaks among children, particularly those with asthma. We previously detected neutralizing antibodies against the predominant EV-D68 B1 clade in the 2014 outbreak in serum collected before the outbreak (2012–2013) from persons 24 months to 85 years of age. We recently detected neutralizing antibodies to the 2014 B1, B2, and D clade viruses in serum collected after the 2014 outbreak (April–May 2017) from 300 children 6 months to 18 years of age. B1 virus neutralizing antibodies were found in 100% of patients, even children born after 2014; B2 in 84.6%, and D in 99.6%. In 2017, titers increased with patient age and were higher than titers in 2012–2013 from comparably aged children. Rate of seronegativity was highest (15.3%) for B2 virus. Multivariate analysis revealed an association between asthma and higher titers against B2 and D viruses. EV-D68 seems to have circulated during 2014–2017.

## Abstract 2

Antibodies to B1, B2, and D clade viruses were detected.

Enterovirus D68 (EV-D68) rose to prominence because of its association with acute flaccid myelitis (AFM) (*1*,*2*) and the US outbreak of severe respiratory disease among children in 2014 (381 cases in Kansas City, Missouri, USA; 1,153 confirmed cases nationally). Severe disease affected children with a history of atopic disease, asthma, or reactive airway disease (*3*–*6*). Although the 2014 EV-D68 outbreak in the United States was caused predominantly by a clade B1 virus, 2 less frequent viruses, clades B2 and D (previously A2), were also detected. In the United States, EV-D68 activity varies year to year and regionally; some areas show a biennial pattern and others do not (*7*), yet EV-D68 seems to be seasonal (primarily late summer through fall).

Before 2014, sporadic small regional/local EV-D68 outbreaks were reported in the United States (*8*) and globally. However, during 2014–2016, EV-D68 was the most frequently reported enterovirus in the United States (*9*). Prevalence of nonoutbreak cases is unclear; however, new B clade viruses emerged in 2012 and 2013 (*10*–*12*), and new B subclade and D clade viruses emerged in 2016–2019 (*12*). In contrast to other US regions, activity in Kansas City was minimal in 2015 (*7*), 2016, and 2017 (R. Selvarangan, unpub. data).

Prospective EV-D68 surveillance has recently been undertaken by the New Vaccine Surveillance Network (NVSN, https://www.cdc.gov/surveillance/nvsn/index.html), which includes Kansas City. NVSN reported an uptick in activity in July and October 2018 (*13*) in not only Missouri (54 detections in Kansas City, clade B3 [*14*]) but also Ohio, Tennessee, Pennsylvania, Texas, Washington, and New York. Clade B3 virus in Kansas City was similar to the virus that caused a 2016 outbreak associated with AFM in nonmidwestern US areas. Nevertheless, increased worldwide attention has led to seroprevalence and genotyping reports from multiple countries (*15*–*20*).

EV-D68 community circulation remains underrecognized because clinically used multiplex respiratory PCR assays do not specifically identify EV-D68. We previously evaluated EV-D68 neutralizing antibodies in serum collected in Kansas City during 2012–2013 from persons 2–85 years of age (*21*). Despite no prior documented EV-D68 outbreaks or outbreaks of EV-D68 compatible illnesses in Kansas City, all samples had neutralizing antibodies to the B1 virus, suggesting EV-D68 circulation before the major outbreak in 2014.

Our goals with this study were to use the same assay that we used previously to evaluate neutralizing EV-D68 antibodies to the 2014 clade B1, B2, and D viruses in serum collected during 2017 from children 6 months to 18 years of age, including those born after 2014, and to examine associations of antibody titers with demographic and medical history factors. This study was approved by the institutional review board at Children’s Mercy Hospital Kansas City.

## Methods

We examined deidentified serum from 300 nonimmunocompromised children 6 months to 18 years of age in Kansas City for EV-D68 neutralizing antibodies. Samples were taken from excess serum after standard-care phlebotomy during April–May 2017 (Appendix). We matched age, sex, and race distributions with those from 2016 Kansas City pediatric census data (*10*). We used the following age groups: 6–35 months of age (n = 76) born after September 2014 (postoutbreak), 36–71 months (n = 51), 72 months–10 years (n = 70), 11–15 years (n = 69), and >15 years (n = 34). We excluded serum from children younger than 6 months because of confounding transplacentally acquired maternal EV-D68 antibodies. We used electronic medical records to document patient age, sex, race, family size, underlying conditions, and number of both hospitalizations and of chest radiographs in the prior 3 years.

The Centers for Disease Control and Prevention (CDC; Atlanta, Georgia, USA) performed serologic testing for this study, using the same microneutralization assay as in our previous study, adapted from a standardized polio antibody assay (*22*,*23*). Three phylogenetically distinct EV-D68 viruses were used: 2014 Missouri 14-18949 (clade B1, GenBank accession no. KM851227); and 2 non-Missouri 2014 strains 14-18952 (clade B2, GenBank accession no. KM851230) and 14-18953 (clade D, formerly A2, GenBank accession no. KM851231). The 2014 detection frequency among US patients was >91% for B1, 7.4% for B2, and <2% for D viruses (*10*).

This EV-D68 microneutralization assay performed at CDC was previously published (*21*,*24*,*25*). In brief, 2-fold serum dilutions, 1:8 to 1:1,024, were combined with 100 cell culture 50% infectious doses of EV-D68 to enable antibody to bind to virus. After 3 hours of incubation, each virus–serum mixture was inoculated onto rhabdomyosarcoma (CCL-136; American Type Culture Collection, https://www.atcc.org) cell monolayers. CDC tested each serum dilution in triplicate against each virus. Each run had known positive control serum (horse antibodies against the Fermon prototype EV-D68 virus); multiple (>4) positive control replicates were distributed across each run. When >7 serum samples were tested in the same run, sample position was randomized via a balanced block randomization scheme. Each run included 2 control plates with no serum or control antibodies; rhabdomyosarcoma cells alone served as a no-virus control. A back-titration virus–control plate was used for each of the 3 EV-D68 strains to confirm the amount of antigen used in each run. A luminescent cell viability kit (ATPlite; Perkin Elmer, http://www.perkinelmer.com) was used to evaluate neutralization, and samples with luminescent activity at a titer of >3 log_2_ (1:8 dilution) were considered to be positive for neutralizing antibodies (*21*,*24*,*25*).

We performed statistical analyses by using Sigmaplot version 12.2 (http://www.sigmaplot.co.uk) for univariate and multivariate analyses; we considered p<0.05 to be significant. We assigned a value of log_2_ 2.5 to seronegative samples. We did not analyze ethnicity and daycare attendance because of incomplete data. Categorical values were analyzed by using the χ^2^ test. We analyzed antibody titers by using the Kruskal-Wallis rank-sum test to determine if overall distributions’ medians significantly differed among groups, and we performed subset comparisons by using the Kolmogorov-Smirnov test. We assessed differences between viruses in each age group by using nonparametric analysis of variance and adjusted for multiple comparisons by using Tukey-Kramer comparisons. To determine whether responses differed between children born after the outbreak and in the year of the outbreak, we used a subset analysis of variance to compare titers for children born in 2014, 2015, and 2016.

We presented comparisons of antibody titer distributions as reverse cumulative distributions (RCD; Appendix). We compared areas under the curve (AUCs) of the RCD curves for each age group among viruses and for each virus among age groups, to represent overall population neutralizing antibody responses by age group (Figure) and by virus (Appendix Figure).

For univariate analysis of demographic and underlying condition data, we used the Mann-Whitney rank sum or the Kruskal-Wallis test, as appropriate. We then used multivariable logistic regression based on binary outcome of high versus low titer to analyze factors significant by univariate analyses. 

## Results

Samples were from 300 patients with a median age of 6.0 years (range 0.5–17.9 years), and 152 (51%) patients were male. Self-reported race/ethnicity from medical records indicated that 200 (66.6%) patients were White, 49 (16.3%) Black, 45 (15.0%) mixed/other, 6 (2%) Asian, 6 (2.0%) Native American, and 1 Micronesian. In total, 33 patients self-reported as Hispanic/Latino and 8 were listed as non-Hispanic/Latino; ethnicity was not available in the medical records for 259 (86.3%) patients. Families can opt out of reporting ethnicity when registering at our institution. Family size averaged 4.4 ± 1.1 members. Overall, the mean number of hospital admissions in the previous 3 years was 1.4 ± 1.1 (range 0–6). Underlying conditions were reported for 130 (43.3%): asthma, 39 (13.0%); neurologic disease, 25 (8.3%); diabetes mellitus, 16 (5.3%); cardiac disease, 15 (5%); renal disease, 13 (4.3%); other lung conditions, 6 (2.0%); blood disorder not cancer, 6 (2.0%); and other disease (hepatic, metabolic, other endocrine), 10 (3.3%).

In all 300 samples, neutralizing antibodies against B1 virus were detected (i.e., >3 log_2_, 1:8 titer) (Table 1). Seropositive rates were lower for B2 (254/300, 84.7%) than for B1 (100%) or D virus 296/300 (98.7%; p<0.001 for each).

**Table 1:** Serum neutralizing antibody positivity and titers for enterovirus D68 clades B1, B2, and D, by patient age group, Kansas City, Missouri, USA, 2017

Age group	No. patients	% Neutralizing antibody positive, median (range) neutralizing antibody titer*		
		B1 clade virus	B2 clade virus	D clade virus
6–35 mo	76	100, 7.83 (5.50–10.5)	76.9, 3.17 (2.5–10.5)	98.1, 5.5 (2.5–9.83)
36–71 mo	51	100, 9.17 (6.17–10.5)	89.8, 6.00 (2.5–10.5)	100, 6.5 (3.5–10.5)
72 mo−10 y	70	100, 9.50 (6.50–10.5)	96.7, 8.83 (2.5–10.5)	99.5, 8.17 (2.83–10.5)
11–15 y	69	100, 10.17 (6.5–10.5)	99.3, 10.17 (2.5–10.5)	100, 10.17 (3.83–10.5)
>15 y	34	100, 10.5 (5.83–10.5)	100, 10.50 (5.5–10.5)	100, 10.5 (4.5–10.5)
Total	300	100, 9.17 (5.5–10.5)	84.6, 7.83 (2.5–10.5)	99.6, 7.50 (2.5–10.5)

*Antibody titers were measured by using the cell viability kit ATPlite (Perkin Elmer, http://www.perkinelmer.com); the titers shown are the log_2_ inverse dilution of the lowest antibody concentration with luminescent activity. Seronegative patients are included.

More samples were seronegative for B2 (n = 76) than for D virus (n = 6). Male patients were overrepresented among those seronegative for B2 virus, 65% (30/46) compared with the overall sample set, for which 48% (122/254) were male (odds ratio 2.029, 95% CI 1.054–3.905; p = 0.03). For the B2 virus, the seronegative rate was higher (25/76, 32.9%) among patients 6–35 months of age (all born after the 2014 outbreak) than among those >36 months of age (21/224, 9.4%) and born before the 2014 outbreak. Two patients 6–35 months of age were seronegative for both B2 and D viruses. Seronegative rates did not differ by race (data not shown).

Median neutralizing titers rose with advancing age (p<0.001; Table 1), but titers among patients 11–15 years of age were similar to those among patients >15 years of age. The overall median titer was highest for B1 viruses (9.17 log_2_, range 5.5–10.5 log_2_) and lowest for D viruses (7.5 log_2_, range 2.5–10.5 log_2_; p<0.001). We found no significant differences in median titers for any of the 3 viruses between children born in 2014, 2015, or 2016 (data not shown). Overall, neutralizing titers did not differ by sex, race, or family size (data not shown).

Patients 8–13 years of age, whose samples were obtained in 2017, would have been 5–10 years of age (the age group previously documented to have had the most severe disease) in 2014 (*21*). The median B1 virus titer for those 8–13 years of age in 2017 is higher (9.83, interquartile range [IQR] 9.5–10.5) than titers for those who were either 8–13 (8.17, IQR 5.83–9.83) or 5–10 (7.83, IQR 4.17–10.5) years of age in 2012–2013 (*21*). Likewise, low titers were more frequent in serum collected in 2012 than in 2017 (Table 2).

**Table 2:** Low versus high neutralizing antibody titers for enterovirus D68 clades B1, B2, and D in serum collected in 2017 compared with titers previously reported from 2012–2013, from patients <18 years of age, Kansas City, Missouri, USA*

Group	Total	No. (%) patients		
B1 clade virus	B2 clade virus	D clade virus
Serum obtained in 2017	300			
Low titer†		0	110 (36.7)	66 (22.0)
High titer		300 (100)	190 (63.3)	234 (78.0)
Serum obtained in 2012–2013 (*18*)	273			
Low titer†		54 (19.8)	117 (42.9)	133 (48.7)
High titer		219 (80.2)	156 (57.1)	140 (51.3)

*Antibody titers were measured by using the cell viability kit ATPlite (Perkin Elmer, http://www.perkinelmer.com). Seronegative patients are included. †Low neutralizing titer defined as <6 log_2_ (<1:64 titer).

RCD curves show differences in the distribution of 5 age groups of patients (Figure); titers of neutralizing antibodies against the 3 viruses targeted in the neutralization assays are expressed along the x-axis. We calculated AUCs and used them for comparative analyses.

**Figure:**
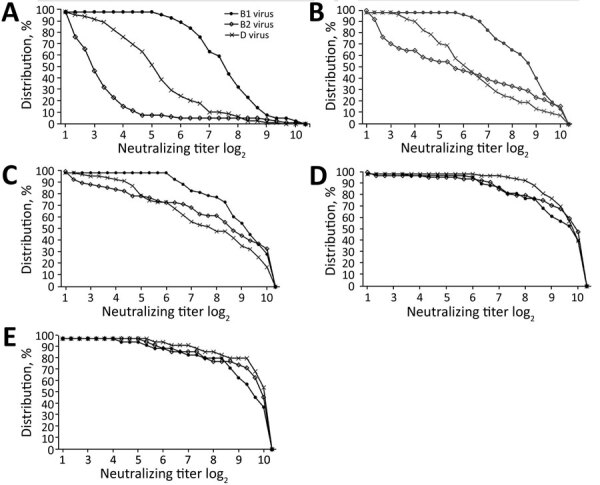
Reverse cumulative distribution (RCD) curves of enterovirus D68 (EV-D68) representing the distribution of neutralizing antibody titers against 3 EV-D68 viruses (clades B1, B2, and D) in serum samples obtained in 2017 from children <18 years of age in Kansas City, Missouri, USA, by patient age group. A) 6–35 months of age; B) 36–71 months of age; C) 72 months–10 years of age; D) 11–15 years of age; E) 16–18 years of age. A titer >3.0 log_2_ was considered positive for neutralizing antibodies. RCDs are curves for which each data point is the proportion of the population with a titer at least as high as the value on the x-axis. The calculated values for each area under curve (AUC) enable comparison of overall immune responses among age groups. Each panel shows 3 RCDs (1 for each virus). Panel A shows that the widest divergence of curves occurred among patients 6–35 months of age, who were born after the 2014 outbreak, suggesting less cross-neutralization among the 3 related viruses in this age group. RCDs become more convergent with each increasing age group. The largest AUCs in each age group are for the B1 predominant 2014 outbreak virus.

The RCDs for the 3 viruses became less divergent with advancing age group. RCD curve AUCs for B1 were larger than those for B2 or for D viruses in the 3 younger age groups, (p<0.001 for each). Within the 2 older age groups, RCD profiles did not differ significantly.

When we evaluated the RCDs for each of the 3 viruses (Appendix Figure) for the 5 age groups, overall RCDs were larger for B1 than for B2 or D viruses (p<0.01). RCD AUCs for each virus became larger with advancing age groups (i.e., smallest for those 6–36 months of age and largest for those >15 years of age). For each virus, RCD differences were most notable for the 3 youngest age groups. Indeed, RCD curves for the 11-15–year age group and the >15 years age group were larger than curves for the 3 younger age groups (p<0.001 for each virus; Appendix Figure).

We performed univariate analysis for associations by using median titer differences. We noted significant differences for patients with asthma (higher median titer 10.17 [IQR 9.17–10.5] vs. 9.17 [IQR 7.83–10.17]; p = 0.001) by univariate analysis (Table 3). Median titers were higher for those who had been hospitalized during the previous 3 years (p = 0.036) but not for the subset admitted specifically for respiratory illness. Other associated factors, but with lower median titers, were chronic nonasthma lung disease (lower median titer 7.17 [IQR 6.75–8.83] vs. 9.5 [IQR 8.09–10.17]; p = 0.01), congenital heart disease (lower median titer 8.17 [IQR 7.17–8.5] vs. 9.5 [IQR 8.17–10.5]; p = 0.02), and a chest radiograph performed in the previous 3 years (p<0.001). Two factors, daycare attendance and ethnicity, were not analyzed because of insufficient patient numbers with data documented in the medical record. For analyzed underlying conditions, no differences were associated with diabetes mellitus, other endocrine disorders, hematologic illness (immune-compromising conditions were excluded), neurologic, renal, hepatic, or metabolic diseases (data not shown)

**Table 3:** Neutralizing antibody titers for enterovirus D68 clades B1, B2 and D, in patients >24 months of age with and without a clinical diagnosis of asthma, Kansas City, Missouri, USA, 2017

Group	No. patients	Neutralizing antibody, median (range)*		
		B1	B2	D
Asthma†	39	9.83 (5.50–10.50)	9.17 (2.50–10.50)	9.17 (3.17–10.50)
No asthma	214	9.50 (5.83–10.50)	8.83 (2.50–10.50)	8.17 (2.50–10.50)
Total	253	9.50 (5.50–10.50)	9.17 (2.50–10.50)	8.83 (2.50–10.50)

*Antibody titers were measured by using the cell viability kit ATPlite (Perkin Elmer, http://www.perkinelmer.com); the titers shown are the log_2_ inverse dilution of the lowest antibody concentration with luminescent activity. Includes seronegative patients. †Asthma as noted by clinician diagnosis in electronic medical record. Because no patients had an asthma diagnosis at <24 mo of age, to balance the age distributions of the nonasthma group with the asthma age group, we excluded nonasthma patients <24 mo of age from this analysis.

Multivariate analysis based on binary categorization (high vs. low titer) and using variables that were significant in univariate analyses revealed persistent significance for a history of asthma (higher titers). However, when we excluded children <24 months of age from the analysis (given that titers are associated with age and no patient in the asthma group was <24 months of age), significantly higher titers persisted for B2 and D viruses only (Appendix Table).

## Discussion

All samples, even from children born after the 2014 outbreak and as young as 6 months, contained EV-D68 neutralizing antibodies to the 2014 outbreak B1 virus. This finding indicates that the outbreak virus, or a closely related EV-D68 strain, circulated in Kansas City from 2014 through 2017. EV-D68 was not detected in the clinical or research laboratory at Children’s Mercy Hospital Kansas City during 2015 or 2017 from research surveillance or clinical samples obtained from children receiving medical care at that hospital. Yet EV-D68 activity in Kansas City after, and presumably during, 2014 may have contributed to the higher titers in samples collected in spring 2017 compared with titers in samples collected in 2012 from children of comparable ages. EV-D68 was detected in 11 routine clinical samples in 2016 and in 255 NVSN research samples collected in 2018 (*13*), but the 2018 detections were all later than the April 2017 date of the samples in our study. Furthermore, excess hospital admissions for severe respiratory disease, particularly intensive care unit admissions, such as had been noted in 2014, were infrequent among children seeking care at our Kansas City institution during 2015–2017 (C.J. Harrison, unpub. data). The only outbreak detected in Kansas City after the 2014 outbreak was caused by a B3 virus in 2018 (*13*) (a national EV-D68 outbreak occurred in 2018 and was associated with increased reports of AFM and emergency department visits/hospitalizations for EV-D68 respiratory illnesses) (*13*).

Our data also confirm age-associated higher titers (e.g., generally increasing median titers and larger RCD curves for the B1 2014 outbreak virus with each increasing age group), the highest being from those in the 2 oldest age groups. Indeed, data for children >11 years of age were remarkably similar to our previously reported data for children of these same ages and to our previous data for adults and elderly persons (*21*). Titers increasing with patient age suggest EV-D68 exposures during nonoutbreak interval years without detected EV-D68 illnesses. If there had been only a single exposure, one might expect antibody titers to peak within 6 months and then decline unless re-exposure occurs (*26*). Nevertheless, higher titers with age could also result in part from increasing EV-D68 antibody specificity over time after initial infection.

Although overall B1 virus titers were lowest for those in the youngest age group (6–35 months), titers were universally >5.0 log_2_ (≈1:64) even in children born since 2014, also suggesting B1 virus circulation sometime during 2015–2017. Alternatively, antibodies elicited by exposure to undetected but related non-B1 viruses may cross-neutralize the targeted viruses (e.g., B1, B2, and D). However cross-neutralization activity may be variable, as suggested by overall differences in titers against B1 virus compared with B2 and D and age-associated differences for each virus. Of note, B2 and D viruses were not detected in Kansas City in 2014 (*8*,*10*). Indeed, the low rates of seronegativity to both B2 and the 2014 D virus in our current and prior studies suggest that antibodies induced by the 2014 B1 virus cross-neutralize B2 and D viruses. Such cross-neutralization seems reasonable given the close relatedness of the B1 and B2 viruses and the less but still relatively close relatedness of the D virus (*27*).

Comparing our data with data from other serosurveys shows similarities and differences. We confirmed our prior data and that of others (i.e., higher overall titers in serosurveys performed soon after outbreak years). A 2011 study from China showed higher neutralizing titers to locally circulating Beijing/2008/01 EV-D68 in postoutbreak 2011 samples compared with preoutbreak 2004 samples, despite few reported EV-D68 illnesses in the Beijing area during 2009–2011 (*16*). Likewise, more recent data from China, Taiwan, the Netherlands, and the United Kingdom show the same pattern of higher neutralization titers in years soon after outbreaks (*20*,*28*).

Similarly, the age-dependent increases in neutralizing titers in this and our previous study (*21*) parallel prior data (*15*,*18*–*20*,*28*,*29*) regardless of any temporal relation to outbreak years. Nevertheless, it was somewhat surprising that titers from 2017 in Kansas City, even in patients born after the 2014 outbreak, were uniformly >1:64 against the 2014 B1 virus outbreak strain. Furthermore, low neutralizing titers (defined as <5 log_2_ or <1:32) were less common in serum collected in 2017 than in our previously reported samples collected from children during 2012–2013 (*21*) against the 2014 major B1 virus (0/300 vs. 54/273 [19.8%]), against B2 virus (110/300 [36.7%] vs. 117/273 [42.9%]), and against D virus (66/300 [22.0%] vs. 133/273 [48.7%]).

Although differences in the assays used by other investigators (e.g., target virus) make comparing absolute titers challenging, our seropositivity rates for patients 6–35 months of age were also higher than those found in other studies before and after the 2014 outbreak (*15*,*17*,*18*,*28*,*29*). It is possible that the modest EV-D68 activity detected in Kansas City in 2016 led to mild or asymptomatic infections in younger children and boosted titers in older children.

We also detected higher titers associated with a history of asthma, but after excluding children too young to have an asthma diagnosis, we found significantly higher titers for only the B2 and D viruses. Nevertheless, asthma was the only underlying condition associated with high titers in multivariate analysis. In 2014, severe EV-D68 respiratory disease occurred in children up to 10 years of age and in populations with atopic disease, asthma, or reactive airway disease, despite what seems to have been the universal presence of neutralizing antibodies, at least in Kansas City children (*8*,*21*). This finding suggests that the mere presence of neutralizing antibodies at a log_2_ titer >3.0 may not be protective against disease, at least in some populations. Protection may occur only if sufficient serum neutralizing antibodies are available. For example, severe respiratory tract disease or AFM is unusual or nonexistent among those in age groups with the highest overall neutralizing titers: adolescents, adults, or the elderly (most with titers >1:256 [i.e., log_2_ >8] in our current and prior studies [*21*]).

Of note, in our current study, neutralizing activity against the non-B1 viruses was higher in children who had asthma as an underlying condition, suggesting an altered response to infection resulting from genetic factors or perhaps to asthma itself (*4*). For example, asthma is associated with enhanced tight junction injury from rhinovirus infection (*30*). Alternatively, immunopathologic responses may play a role, as can be seen in the influenza cotton rat model (*31*). Serum neutralizing antibodies also may not correlate best with protection. For example, T-cell activity or mucosal antibodies may be more protective than serum antibodies (*32*), or perhaps antibodies to certain epitopes are crucial, as suggested in an EV-D68 mouse model in which monoclonal antibodies were more effective than convalescent polyclonal antibodies in intravenous immunoglobulin preparations (*33*).

Unlike one previous study (*29*), we did not find family size to be associated with seropositivity. We could not evaluate our prior observation of lower titers in Hispanic patients (*21*) because of low numbers of self-reports of ethnicity (41/300). Similarly, we could not analyze effects of daycare (data available for only 36/300).

Limitations of our study include a study design that used salvaged samples and a retrospective chart review. Because we tested for neutralizing antibodies against only 3 EV-D68 strains, patterns of neutralizing activity against other EV-D68 strains could differ. However, we did test for the 2014 B1 clade strain known to have circulated in Kansas City as well as B2 and D viruses. EV-D68 activity in Kansas City during 2016 (clade unknown) was low but could have boosted titers. Indeed, we also noted EV-D68 activity in 2018 (B3) and 2020 (D). Age ranges for our pediatric groups could be considered arbitrary; the age groups we used were similar to those used in our previous study, except we added children 6–35 months of age, paralleling other reports (*3*). The racial and age distributions of our population matched those of Kansas City census data and, therefore, might not be generalizable to other geographic areas. That said, these distributions closely mirrored those of the United States as a whole during 2015–2017. Last, the numbers of patients with each underlying condition were relatively small, so we may not have had the power to detect associations (e.g., higher titers to B1 virus in those with asthma).

In conclusion, we detected neutralizing antibodies to the dominant 2014 B1 clade EV-D68 virus at titers >1:64 for all 300 serum samples from children in 2017, a time frame with little documented EV-D68 activity since the 2014 outbreak. In the same samples, overall titers to the less frequently detected B2 and D viruses were lower. Titers increased with increasing age. Titers against B2 and D virus were higher in those with asthma. Our findings support the concepts that an unusual host–virus interaction of EV-D68 occurs in children with asthma and that EV-D68 can cause disease despite the presence of at least some neutralizing antibodies. 

Appendix Supplemental methods and results for study of neutralizing enterovirus D68 antibodies in children after 2014 outbreak, Kansas City, Missouri, USA.
