# Supplementary material for: Neutralizing Enterovirus D68 Antibodies in Children after 2014 Outbreak, Kansas City, Missouri, USA
Source: Emerg Infect Dis. 2022 Mar;28(3):539–47. doi: 10.3201/eid2803.211467 (PMC8888215; doi:10.3201/eid2803.211467)
Supplement: Appendix — Supplemental methods and results for study of neutralizing enterovirus D68 antibodies in children after 2014 outbreak, Kansas City, Missouri, USA. [file 21-1467-Techapp-s1.pdf]

Article DOI: https://doi.org/10.3201/eid2803.211467

Neutralizing Enterovirus D68 Antibodies in Children after 2014 Outbreak, Kansas City, Missouri, USA

Appendix

Selection of serum samples

The selection process involved construction of a grid with 300 slots that matched the 2016 US Census for age group, gender, and race of the Kansas City region. Sera were then salvaged from consecutive standard of care clinical samples in the clinical laboratory that also had sufficient volume for the assay until each slot was filled.

Rationale for RCD analysis of serology results

RCDs can be directly compared via area under the curve (AUC) calculations as a quantitative measures of population seroresponse, e.g., the RCDs in one population can be compared among different age groups or to those in our 2019 EV-D68 serology manuscript in EID. The RCD also provides a visual curve of proportions of the population that have a titer at least equivalent to each X axis value. X axis values encompass the range of titers detected in the assay. Thus, RCDs allow population-based or target-virus-based comparisons, both visually and statistically (e.g., AUC differences).

As noted in 1995 (1), RCDs are visual representations that allow "comprehension of the complete distribution of the values, limited only by the quality and resolution of the graph. Whereas numerical summaries and statistical tests of antibody data address for the most part the equality of the mean or median values, these graphs display all the differences in antibody distributions. This technique allows direct comparison of data sets, which is not convenient with other graphic tools such as histograms or stem-and-leaf displays. It also conveys the importance of each data point, whereas box plots display more of a visual summary for the majority of data points. The RCD plots also meet the criteria for techniques used for exploratory data analyses,

i.e., they need no prior assumptions about the data distribution; they display outliers without distortion; they highlight important aspects of the data; and they allow comparison of complete distributions of antibody data” (*1*).

Thus, RCD curves have been proposed as useful and perhaps the best tools for exploration and presentation of serological data in selected populations to allow display of the full distribution of data.

Reference

1. Reed GF, Meade BD, Steinhoff MC. The reverse cumulative distribution plot: a graphic method for exploratory analysis of antibody data. Pediatrics. 1995;96:600–3. PubMed https://doi.org/10.1542/peds.96.3.600

Appendix Table. Low versus high neutralizing titers in serum collected in 2017 from children >2 years of age with (n = 39) and without (n = 214) a history of asthma*

Virus clade, neutralizing titer	Asthma, no. (%)	No asthma, no. (%)	Odds ratio (95% CI), p value
B1			1.076 (0.505–22.857), p = 1.000
Low†	0	2 (0/9)	
High	39 (100)	212 (99.1)	
B2			0.3662 (0.1466–0.9147), p =0.036
Low	6 (15.4)	71 (33.2)	
High			
D			0.2241 (0.0664–0.7562), p = 0.007
Low	3 (7.7)	58 (27.1)	
High	36 (92.3)	156 (72.9)	

*Clinical history of asthma noted in the electronic medical record.
†Low neutralizing titer defined as <6 log2 (<1:64 titer).

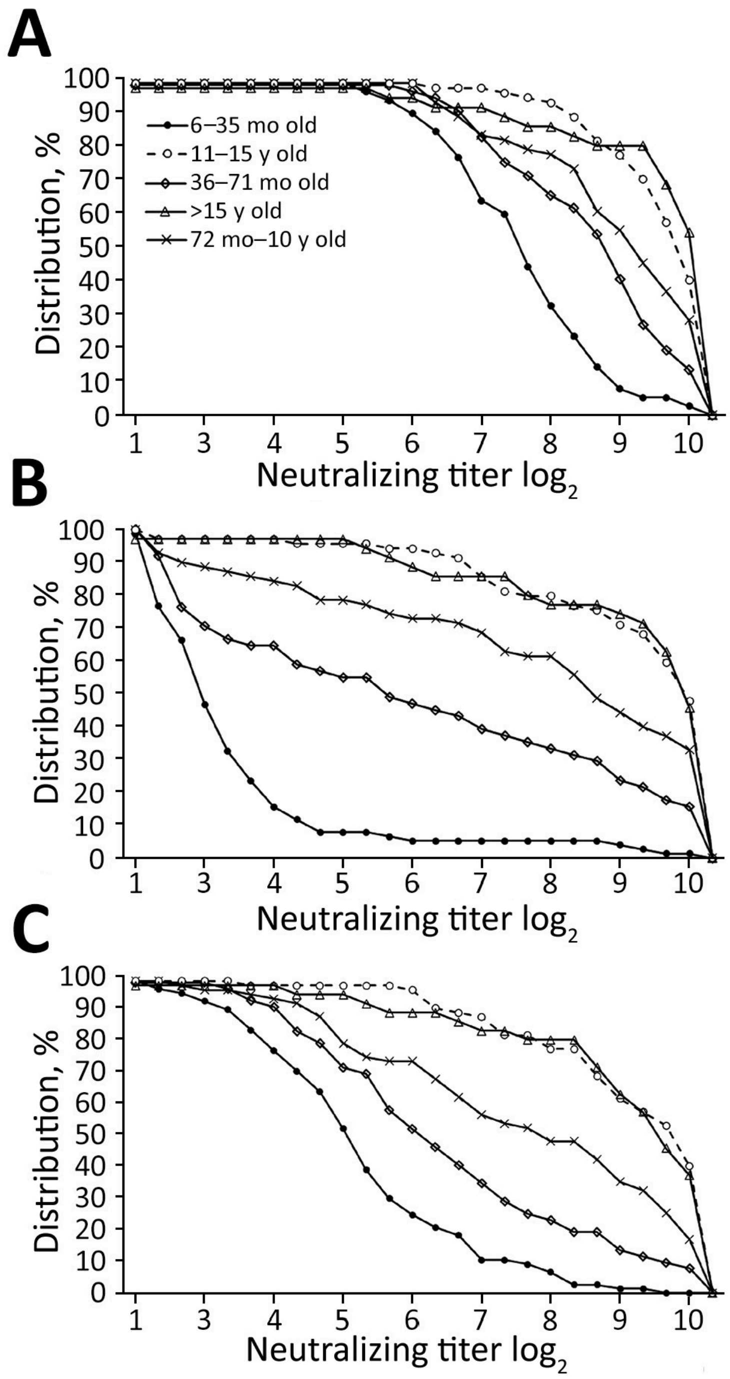

Appendix Figure. Reverse cumulative distribution (RCD) curves for three enterovirus D68 (EV-D68) viruses used as targets for serum neutralizing antibody assays. RCDs are curves for which each data point is the proportion of the population with a titer at least as high as the value on the *x* axis. Sera were obtained in 2017 from children under 18 years of age in Kansas City, Missouri, USA. A titer >3.0 log2 was considered positive for neutralizing antibodies. Each of the three panels has five RCDs, one RCD for each age group. The calculated values for each area under curve allow comparison of overall immune responses among age groups. AUCs are most divergent between age groups for the B2 clade virus, an

infrequently detected virus, with the younger age groups having smaller AUCs (curves shifted to the right). AUCs are largest (shifted to the right) for the B1, the major 2014 outbreak virus, even for the 6–35-month-olds who were born after the outbreak. A) 14–18949 (MO/49) B1 clade virus. Overall titers increased with increasing age. The lowest titer distribution and smallest RCD curve AUC was in the 6–35-month-olds, (p<0.001). The 36–71-month-old and 72 month to 10-year-old overall titer and RCD curves' AUCs were not significantly different. The 11–15-year-old and >15-year-old overall titers and RCD curve AUCs did not differ significantly. However, the older two age groups were each overall higher and RCDs curve AUCs larger than those of 36–71-month-olds and 72 month to 10-year-olds (p<0.01 for each). B) 14–18952 (IL/52) B2 clade virus. Overall titers also increased with increasing age. The lowest titer distribution and smallest RCD curve AUC were also in the 6–35-month-olds the B2 virus, (p<0.001). The 36–71-month-olds' overall titer distribution was lower and RCD curves AUCs were significantly smaller than each older age group, (p<0.01 for the 72 month to 10 years-olds, and p <0.001 for each of the oldest two age groups). The 72 month to 10-year-old overall titer distribution was lower and RCD AUCs were significantly smaller than for the 11–15-year-olds and for the >15-year-olds (p<0.01 each). The oldest two age groups did not differ significantly. C) 14–18953 (KY/53) D (previously A2) clade virus. Differences by age group were similar to those seen against the B1 virus, but to a lesser extent. The lowest titer distribution and smallest RCD curve AUC were again in the 6–35-month-olds for the C virus, (p<0.01 versus the 36–71-year-olds, and p<0.001 versus the other four groups). The 36–71-month-old's overall titer distribution and RCD curves AUCs did not differ significantly from those of the 72 month to 10 years-olds. RCDs and AUCs for the oldest two age groups also did not differ significantly. However, the older two age groups were each overall higher and RCDs curve AUCs larger than both those of 36–71-month-olds and of 72 month to 10-year-olds (p<0.001 for each).
